# Characterization of a copper responsive promoter and its mediated overexpression of the xylanase regulator 1 results in an induction-independent production of cellulases in *Trichoderma reesei*

**DOI:** 10.1186/s13068-015-0249-4

**Published:** 2015-04-14

**Authors:** Xinxing Lv, Fanglin Zheng, Chunyan Li, Weixin Zhang, Guanjun Chen, Weifeng Liu

**Affiliations:** State Key Laboratory of Microbial Technology, School of Life Science, Shandong University, No.27 Shanda South Road, Jinan, 250100 Shandong People’s Republic of China

**Keywords:** *Trichoderma reesei*, Copper responsive promoter, XYR1, Cellulase, Carbon catabolite repression

## Abstract

**Background:**

*Trichoderma reesei* represents an important workhorse for industrial production of cellulases as well as other proteins. The large-scale production is usually performed in a substrate-inducing manner achieved by a fine-tuned cooperation of a suite of transcription factors. Their production and subsequent analysis are, however, often either difficult to manipulate or complicated by the concomitant production of other inducible proteins. Alternatives to control gene expression independent of the nutritional state are thus preferred in some cases to facilitate not only biochemical studies of proteins but also genetic engineering of the producer.

**Results:**

We identified a copper transporter encoding gene *tcu1* (jgi:Trire2:52315) in *T. reesei*, the transcription of which was highly responsive to copper availability. Whereas excess copper repressed the expression of *tcu1* from *T. reesei*, eliminating copper addition in the medium resulted in a high-level transcription of *tcu1*. The usefulness of the system was further illustrated by the high-level expression of specific cellulases driven by the *tcu1* promoter in *T. reesei* when cultivated on D-glucose or glycerol as the sole carbon source. A recombinant *T. reesei* strain, which overexpressed the main transcription activator of hydrolases (xylanase regulator 1) under the control of *tcu1* promoter, was found to be relieved from the carbon catabolite repression and thus displayed a constitutive cellulase expression. Moreover, the amount and activities of cellulases produced by this strain on glycerol or glucose fully recapitulated those of the parental strain produced on Avicel.

**Conclusion:**

Expression of *T. reesei tcu1* gene was tightly controlled by copper availability, and a homologous protein expression system was developed based on this promoter. Deregulation of XYR1 (xylanase regulator 1) mediated by the *tcu1* promoter not only overcame the carbon catabolite repression of cellulases but also resulted in their full expression even on the non-inducing carbon sources.

## Background

The ascomycete *Trichoderma reesei* (teleomorph *Hypocrea jecorina*), a saprophytic filamentous fungus, is one of the most prolific lignocellulosic enzyme producer in industry. The secreted cellulase mixture synergistically acts upon the insoluble cellulosic materials to achieve the efficient conversion to fermentable sugars [1]. Up to 100 g/l of secreted proteins under optimal culture conditions has been reported for *T. reesei* strains [2]. High-yield production of the bulk of plant cell wall-degrading machinery in *T. reesei* is, however, dependent on the induction by insoluble substrates that include cellulose, hemicellulose, or mixtures of plant polymers. Considering the ease of manipulation and the complication of separating enzymes from insoluble plant cell wall materials, soluble inducing substrates are usually preferred or required. These soluble inducers are, however, either non-economic (for example, sophorose) or with low efficiency (for example, lactose).

In *T. reesei*, most of the cellulase genes are coordinately controlled by a suite of transcription factors [3]. Whereas CRE1 is the main transcription factor mediating carbon catabolite repression (CCR) of cellulase gene expression, xylanase regulator 1 (XYR1) is absolutely necessary for activating the expression of most of cellulases/hemicellulases. Lack of XYR1 has been shown to eliminate cellulase induction by almost all inducers [4]. Regulation of *xyr1* expression has thus been considered to exert a significant impact on the ability of *T. reesei* to produce the various hydrolytic enzymes though the exact mechanism remains elusive [5,6]. Two seemingly opposing but not necessarily exclusive assumptions exist regarding the mechanism by which XYR1 may govern cellulase expression. Studies by Mach-Aigner *et al*. have indicated that posttranslational modification of XYR1 constitutes a major activating aspect of its activation [5], whereas other studies suggested that a strongly elevated transcription level of *xyr1* is important for hyperproduction of cellulases under inducing conditions [6,7].

Developments over the past nearly 30 years have also established *T. reesei* as an incredibly useful host for large-scale protein production, largely based on the regulatory elements of the gene encoding the most abundant secreted cellulase component, cellobiohydrolase I (CEL7A) [8,9]. On the other hand, the need to further improve the performance of the native cellulase system of *T. reesei* also requires its being augmented by the introduction of heterologous enzymatic activities or improved variants of native enzymes. Problems may arise, however, with these inducible systems due to the unselective expression of endogenous cellulase components leading to contamination of target proteins for further analysis. Alternative promoters with ability to control gene expression by both activation and repression independent of nutritional state are thus desirable in these cases. In *T. reesei*, many constitutive promoters, most of which are from genes participating in glucose metabolism, have been reported to drive gene expression including specific cellulase and xylanase encoding genes on glucose [10]. These promoters are useful, but not ideal, because they are highly influenced by the nutritional state of the culture and their behavior on carbon sources other than glucose has not been systematically studied. More recently, promoters of the high-affinity copper transporter encoding genes have been used successfully in several fungi to drive high-level gene expression [11,12]. Nevertheless, the existence of such copper transporters and the potential of using their promoters to tightly control gene expression in *T. reesei* have not been examined yet.

In this paper, we developed a copper-responsive expression system based on the promoter of a *T. reesei* copper transporter gene *tcu1*. The success of this system was further demonstrated by the tunable expression of proteins including two cellulases (CEL7A and CEL7B) on glucose. Finally, we showed that overexpression of *xyr1* alone driven by P_*tcu1*_ resulted in the full production of cellulases on glucose up to a level comparable to those produced on cellulose.

## Results

### *T. reesei tcu1* gene expression is controlled by copper availability but independent of carbon sources

**Figure 1:**
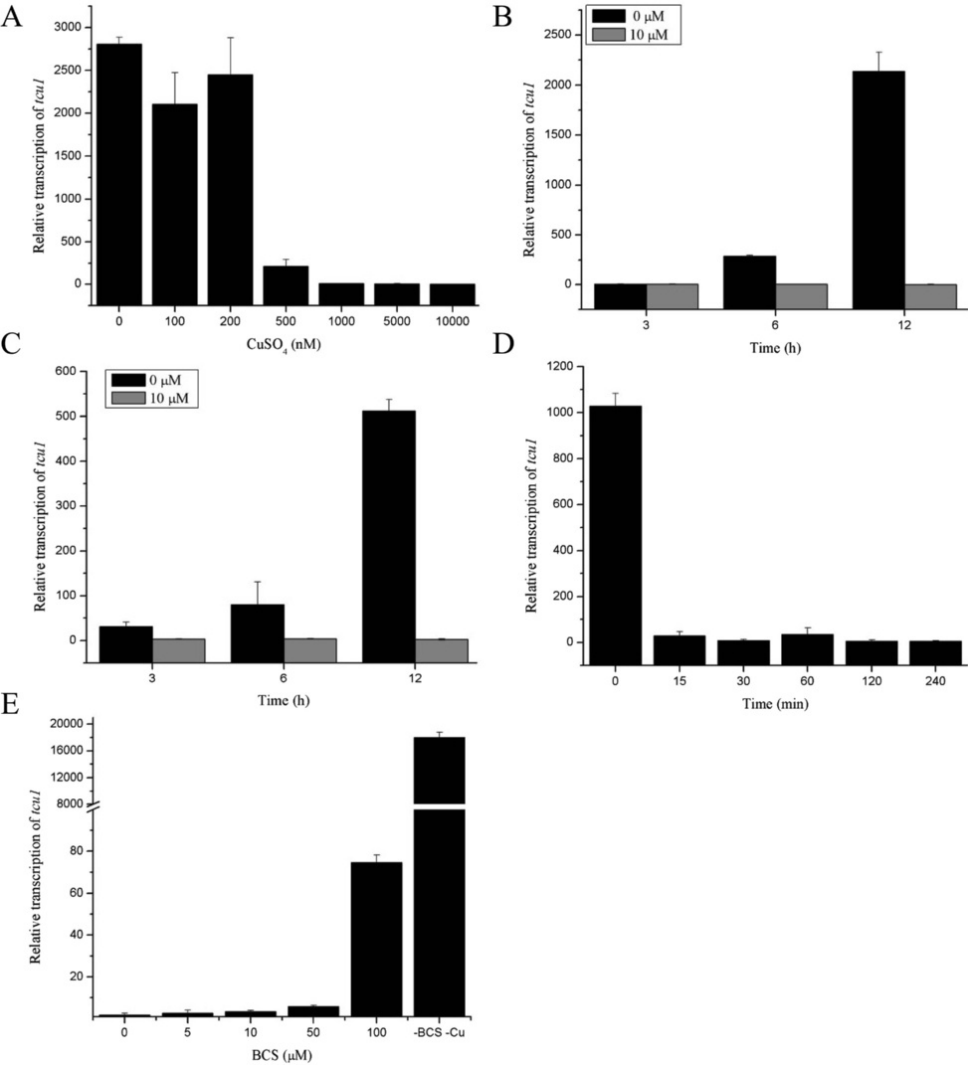
Expression of the *tcu1* gene was influenced by copper availability in *T. reesei*. **(A)** qRT-PCR analysis of the endogenous *tcu1* mRNA after strain QM9414 was cultured for 12 h with 1% glucose as carbon source and with different concentrations of CuSO_4_. A significant difference (*P* < 0.05) was calculated with a Student’s *t* test for the transcription of *tcu1* between concentrations from 0 to 200 nM of copper and those above 500 nM. Levels of endogenous *tcu1* mRNA from strain QM9414 cultured in the presence of 10 μM CuSO_4_ with 1% glycerol **(B)** and 1% Avicel **(C)** as the sole carbon source, respectively. A significant difference (*P* < 0.05) existed in the transcription of *tcu1* at 12 h. **(D)** The kinetics of turnoff of *tcu1* after 10 μM CuSO_4_ addition. A significant difference (*P* < 0.05) existed in the transcription of *tcu1* after adding copper for 15 min compared to 0 min. **(E)** qRT-PCR analysis of *tcu1* mRNA after the indicated concentrations of BCS were added to a QM9414 culture for 12 h in the presence of 1 μM CuSO_4_. A significant difference (*P* < 0.05) existed for the transcription of *tcu1* with and without 100 μΜ BCS. Error bars are the SD from three biological replicates.

Copper transporter genes are sensitive to the environmental copper levels to tightly control the uptake of copper [13,14]. To investigate whether such an ortholog of copper transporter also exists in cellulolytic *T. reesei*, we first searched the genome of *T. reesei* for homologues of the *Neurospora crassa* protein TCU-1 (NCU00830) and a putative copper transporter (jgi:Trire2:52315) was identified. We then examined the expression of *tcu1* in a *T. reesei* strain QM9414 cultured at 12 h in the presence of different concentrations of copper sulfate (0 to 10,000 nM). The results showed that *tcu1* was highly transcribed when less than 200 nM of copper sulfate was added (Figure 1A). The transcription was dramatically decreased by 500 nM of copper sulfate and was almost abolished by copper sulfate above 1 μM (Figure 1A). Importantly, the same response was also observed when the same strain was cultured on glycerol or even cellulose (Figure 1B,C). To determine the kinetics of turnoff of *tcu1* by CuSO_4_, we analyzed the effect of addition of 10 μM copper to a preculture on 1% glucose on the transcription of *tcu1* over time in strain QM9414. As shown in Figure 1D, turnoff of *tcu1* was rapid and robust with the transcription of *tcu1* being shut down as early as 15 min after addition of CuSO_4_ and last for up to 4 h. To further investigate whether the repressed *tcu1* could be directly turned on, we applied different concentrations of a copper chelator bathocuproinedisulfonic (BCS) acid to a QM9414 culture in the presence of 1 μM Cu^2+^ (Figure 1E). The result showed that, as compared with the transcription in the absence of copper, BCS at a concentration up to 100 μM only partially activated the transcription of *tcu1*. Taken together, these data indicated that the transcription of *tcu1* from *T. reesei* was very sensitive to the environmental copper and that the promoter of *tcu1* may well be used for the regulated expression of target genes.

### Copper-responsive expression of the green fluorescent protein by the promoter of *tcu1*

**Figure 2:**
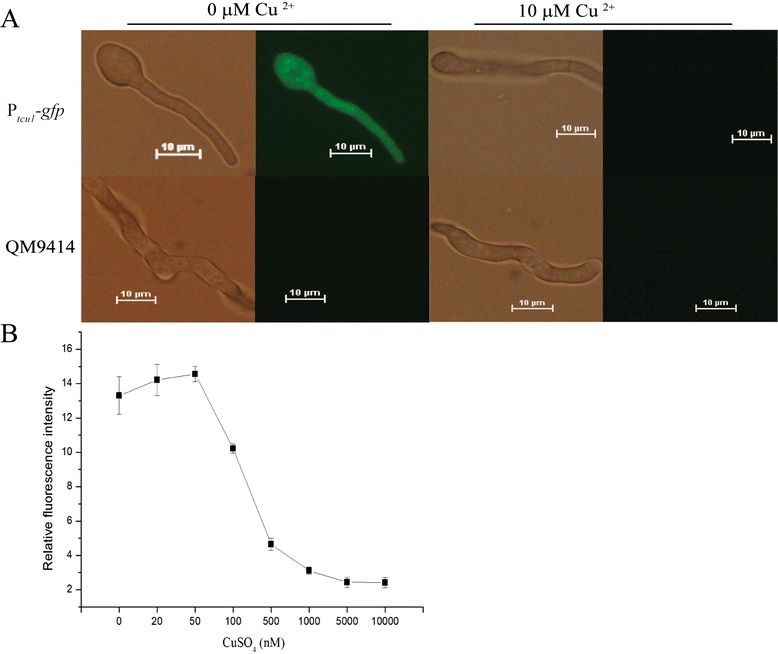
Fluorescent analysis of the recombinant P_*tcu1*_-*gfp* strain. **(A)** Microscopic analysis of the GFP signal of the P_*tcu1*_-*gfp* and QM9414 strains with and without CuSO_4_ addition for 16 h. **(B)** Relative fluorescence intensity of the P_*tcu1*_-*gfp* mycelia in response to the indicated concentrations of CuSO_4_. Error bars are the SD from three biological replicates.

In order to confirm that the promoter of *tcu1* (P_*tcu1*_) could be used to regulate the expression of a heterologous protein, a recombinant QM9414 strain (P_*tcu1*_*-gfp*) expressing the green fluorescent protein (GFP) under the control of P_*tcu1*_ was constructed. The transformant P_*tcu1*_*-gfp* was highly fluorescent when cultured in the absence of CuSO_4_ on 1% glucose for 12 h, whereas no fluorescence was observed when 10 μΜ of CuSO_4_ was included (Figure 2A). Mycelia fluorescence intensity was quantitatively analyzed to reflect the expression level of GFP driven by P_*tcu1*_ at different concentrations of copper sulfate (Figure 2B). In accordance with the results of the expression of the endogenous *tcu1* gene, the fluorescence intensity was the highest when CuSO_4_ was present at a concentration lower than 50 nΜ. Increasing concentrations of CuSO_4_ decreased the fluorescence intensity with a 90% decrease at 500 nΜ, and the fluorescence was nearly extinguished by copper concentrations above 5 μΜ. These data demonstrated that the heterologous protein level expressed under the control of P_*tcu1*_ correlates with copper availability and that P_*tcu1*_ behaves in a highly responsive manner to exogenous copper.

### Homologous expression of two cellulases by P_*tcu1*_

**Figure 3:**
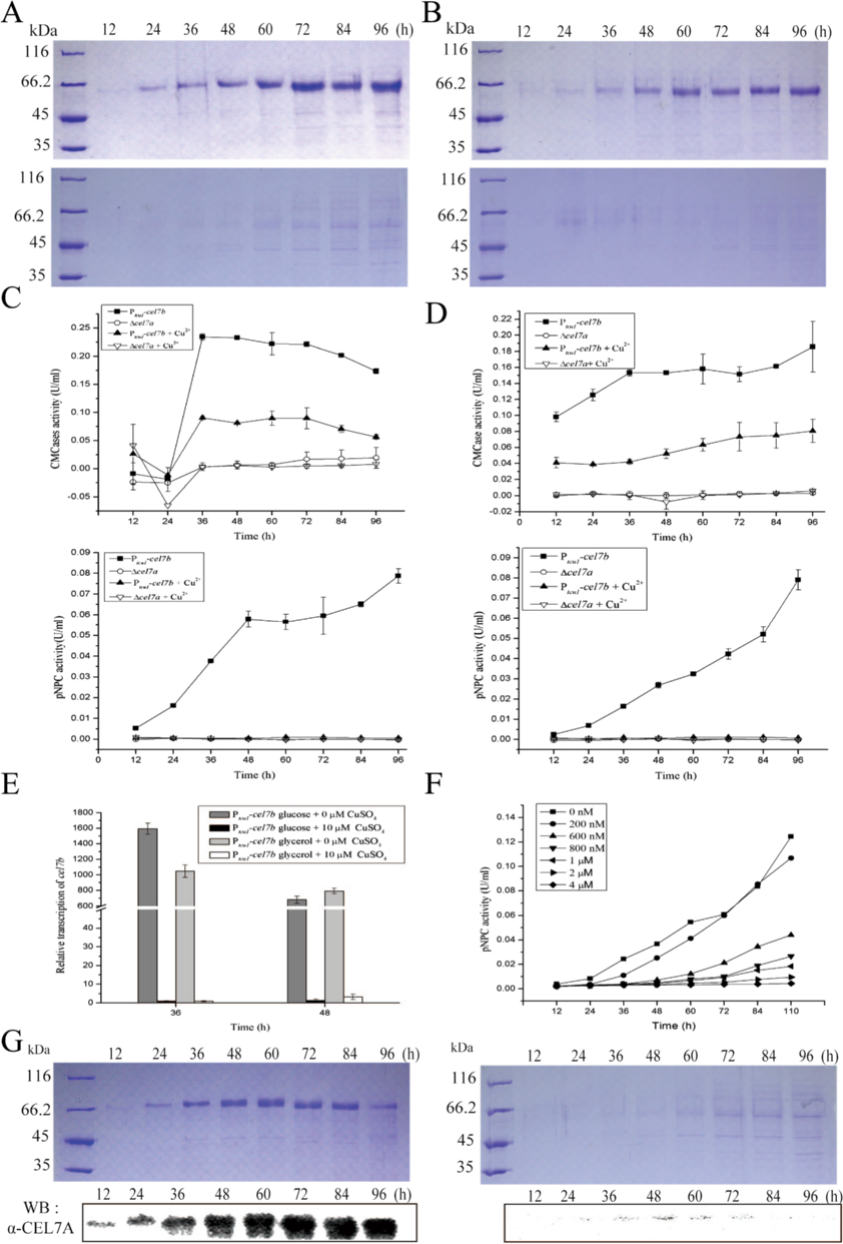
Homologous expression of CEL7A and CEL7B mediated by P_*tcu1*_. SDS-PAGE analysis of the extracellular proteins of P_*tcu1*_-*cel7b* grown on 1% (wt/vol) glucose **(A)** or glycerol **(B)** with (lower panel) or without (upper panel) addition of CuSO4. The extracellular CMC and pNPC hydrolytic activities of P_*tcu1*_-*cel7b* and its parental strain Δ*cel7a* grown on 1% (wt/vol) glucose **(C)** or glycerol **(D)**. Error bars are the SD from two biological replicates. **(E)** qRT-PCR analysis of the c*el7b mRNA* of P_*tcu1*_ *-cel7b* cultured with 1% (wt/vol) glucose or 1% (wt/vol) glycerol in the presence or absence of 10 μM CuSO_4_. A significant difference (*P* < 0.05) existed for the transcription of *cel7b* with and without 10 μΜ Cu^2+^. Error bars are the SD from three biological replicates. **(F)** The extracellular pNPC hydrolytic activities of P_*tcu1*_-*cel7b* in the presence of different concentrations of copper sulfate. **(G)** SDS-PAGE and Western blot analysis of the extracellular production of CEL7A by P_*tcu1*_-*cel7a* grown on 1% (wt/vol) glucose with (right panel) or without (left panel) addition of CuSO_4_. Equal amount of culture supernatant relative to biomass was measured for all the assays.

To further test the potential of developing a P_*tcu1*_-based expression system in *T. reesei*, two cellulase genes including cellobiohydrolase encoding gene (*cel7a*) and endocellulase encoding gene (*cel7b*) were chosen for testing the expression. The respective expression plasmids were transformed into a Δ*cel7a* strain to avoid the potential contamination of endogenous CEL7A, and the extracellular protein production as well as the hydrolytic activity was followed during growth on 1% D-glucose or 1% glycerol. The growth rate was similar with or without CuSO_4_ (data not shown). As shown in Figure 3A,B,G, a significant amount of CEL7B and CEL7A was detected in the culture supernatant of transformants P_*tcu1*_*-cel7b* and P_*tcu1*_*-cel7a*. The produced CEL7B and CEL7A was not due to an endogenous expression since no corresponding protein bands could be detected in the parental Δ*cel7a* strain on glucose or glycerol regardless of the presence or absence of copper (data not shown). Moreover, these proteins were absent from the culture supernatant of the respective transformants when 10 μM CuSO_4_ was included, suggesting the expression was specifically responsive to the copper. Further examination of the *cel7b* transcripts by quantitative real-time PCR (qRT-PCR) revealed that, while the expression of the endogenous *cel7b* was below a detectable level, a significant induced transcription was observed for *cel7b* in the P_*tcu1*_-*cel7b* strain in the absence of copper, which was dramatically decreased by 10 μM copper (Figure 3E). The produced CEL7A was verified by Western blot (Figure 3G). Analysis of the hydrolytic activities verified that the produced CEL7B displayed significant carboxymethylcellulose (CMC) and *p*-nitrophenol-D-cellbioside (pNPC) hydrolytic activities which were strictly dependent on copper availability (Figure 3C,D,F), whereas hardly any pNPC hydrolytic activity was detected from the culture supernatant of P_*tcu1*_*-cel7a* (data not shown), indicating that CEL7A produced under this condition may not adopt a correct conformation. Thus, the *T. reesei tcu1* promoter can be used for copper-controlled expression of both homologous and heterologous proteins.

### P_*tcu1*_-mediated overexpression of XYR1 recapitulates fully induced expression of cellulases on non-inducing carbons

**Figure 4:**
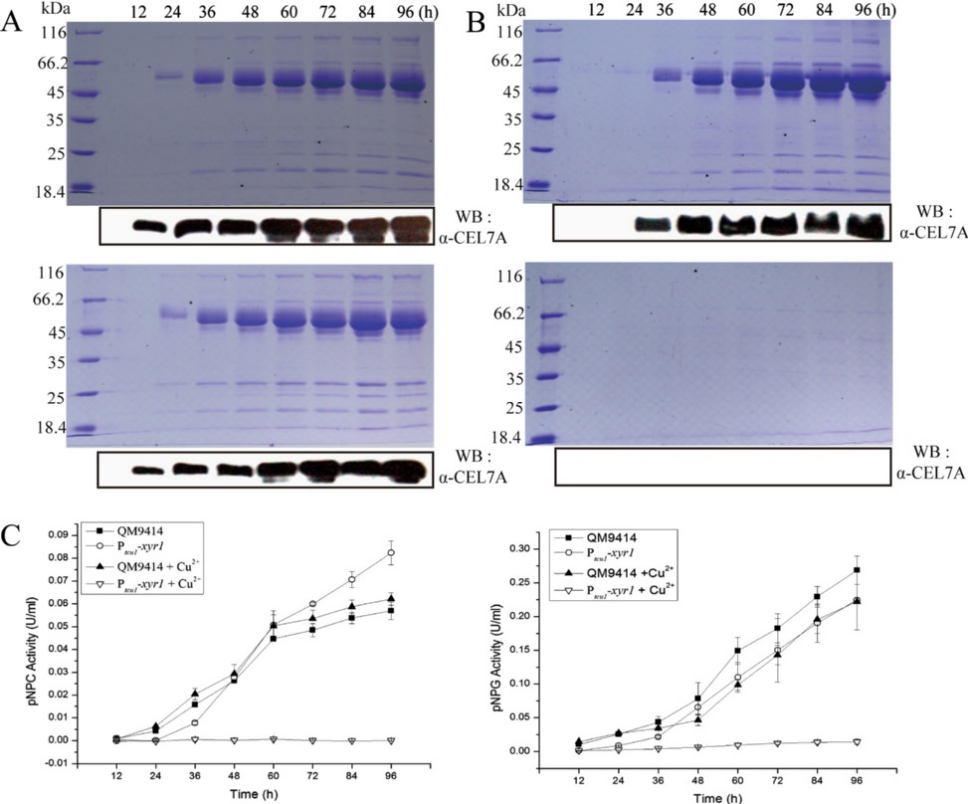
P_*tcu1*_ *-xyr1* recovered cellulase gene expression on induction by 1% Avicel. SDS-PAGE and Western blot analysis of the secreted proteins in the culture supernatant of QM9414 **(A)** and P_*tcu1*_-*xyr1* **(B)** strains grown on 1% (wt/vol) Avicel with (lower panel) or without (upper panel) addition of CuSO_4_. **(C)** pNPC and pNPG hydrolytic activities of the culture filtrates from QM9414 and P_*tcu1*_-*xyr1* strains induced with 1% (wt/vol) Avicel and cultured in the presence or absence of CuSO_4_. A significant difference (*P* < 0.05) was detected for the culture filtrates of P_*tcu1*_ *-xyr1* after 60 h with and without 10 μM CuSO4. Equal amount of culture supernatant relative to biomass was measured for all the assays. Error bars are the SD from at least two biological replicates.

**Figure 5:**
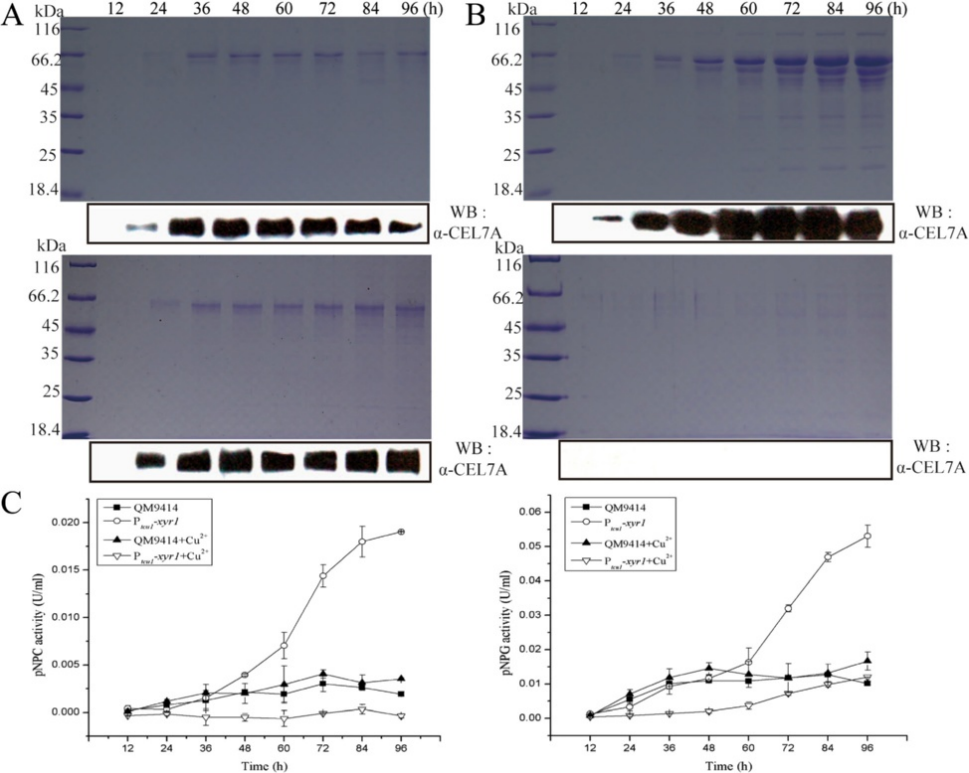
P_*tcu1*_ *-xyr1* recovered cellulase gene expression on induction by 1% lactose. **(A)** SDS-PAGE and Western blot analysis of the secreted proteins in the culture supernatant of QM9414 **(A)** and P_*tcu1*_-*xyr1* **(B)** strains grown on 1% (wt/vol) lactose with (lower panel) or without (upper panel) addition of CuSO_4_. **(C)** pNPC and pNPG hydrolytic activities of the culture filtrates from QM9414 and P_*tcu1*_-*xyr1* strains induced with 1% (wt/vol) lactose and cultured in the presence or absence of CuSO_4_. A significant difference (*P* < 0.05) was detected for the culture filtrates of P_*tcu1*_ *-xyr1* after 72 h without copper addition compared with those of QM9414. Error bars are the SD from at least two biological replicates.

XYR1 has been established as the major regulator for the cellulolytic response to inducing substrate including Avicel or lactose in *T. reesei* [4,5,15]. Strongly increased basal expression of *xyr1* has thus been found to correlate with the hyperproduction of cellulases though that still entails the presence of inducers [16,17]. To probe the effect of copper-controlled expression of *xyr1* on cellulase production, we constructed a recombinant strain expressing *xyr1* under the control of P_*tcu1*_. After transformation of the Δ*xyr1* strain, several independent transformants were isolated based on their recovered ability to form hydrolytic halos on Avicel plate (data not shown). One transformant with the largest halo (P_*tcu1*_-*xyr1*) was chosen for further analysis. To first test whether the P_*tcu1*_*-*driven expression of *xyr1* could recover the cellulolytic phenotype of the Δ*xyr1* strain, the proteins induced by 1% Avicel in the culture filtrate were resolved by sodium dodecyl sulphate-polyacrylamide gel electrophoresis (SDS-PAGE). Whereas cellulases were efficiently induced in the strain QM9414 regardless of the addition of copper or not (Figure 4A), comparable levels and patterns of induced cellulases were only observed for P_*tcu1*_-*xyr1* without addition of copper (Figure 4B), indicating that the induced production of cellulases in the Δ*xyr1* strain was absolutely dependent on the copper-controlled expression of *xyr1*. Analysis of the extracellular pNPC and *p*-nitrophenyl-β-D-glucopyranoside (pNPG) hydrolytic activities demonstrated that, in accordance with the SDS-PAGE results, the hydrolytic activities as displayed by the culture supernatant of P_*tcu1*_-*xyr1* without addition of CuSO_4_ were overall comparable to those of QM9414 (Figure 4C). Hardly any hydrolytic activities were detected for P_*tcu1*_-*xyr1* in the presence of 10 μΜ CuSO_4_. Efficient production of cellulases by P_*tcu1*_-*xyr1* was also observed upon incubation with lactose, and the detected hydrolytic activities of the culture filtrate were significantly higher than those of QM9414 under the same culture conditions after 72 h of induction (Figure 5).

**Figure 6:**
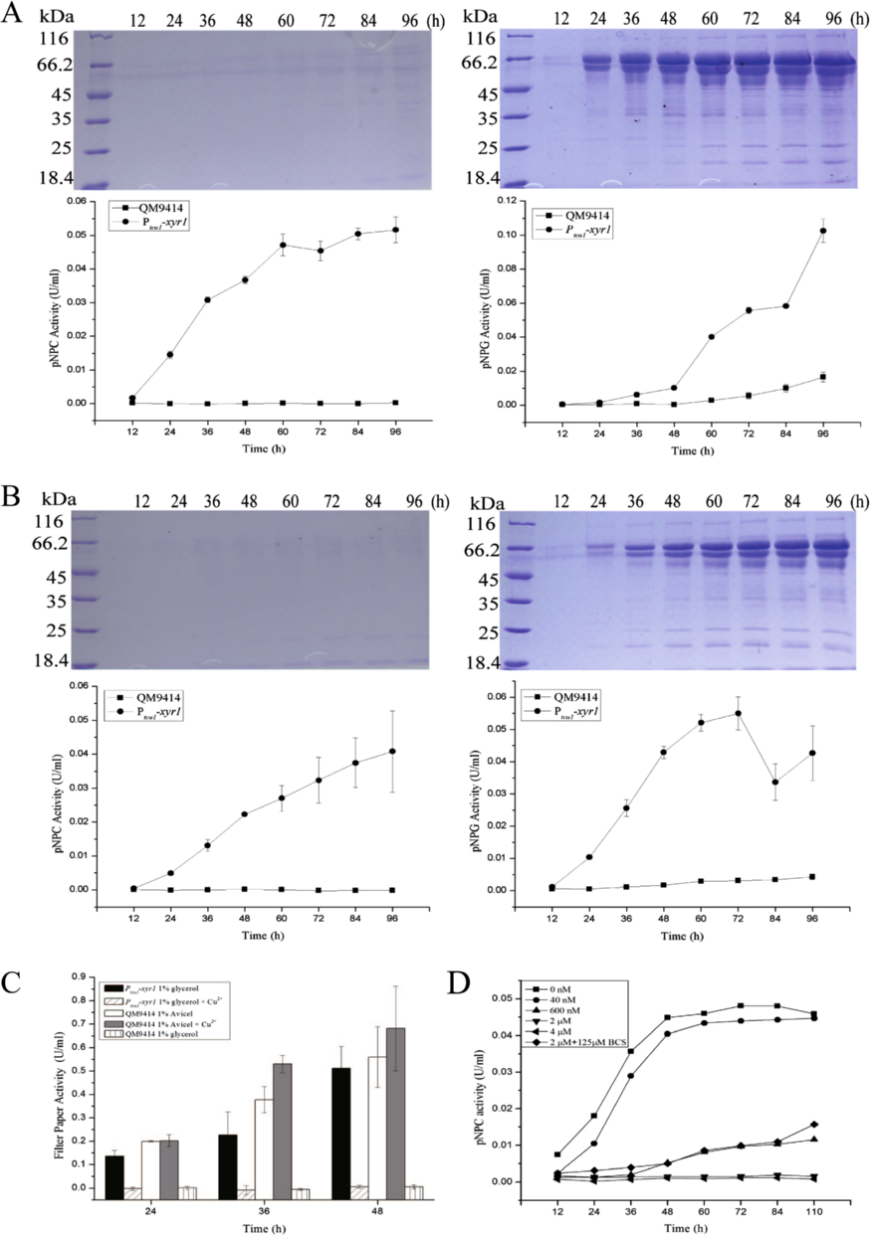
P_*tcu1*_ *-xyr1* displayed a full production of cellulases on non-inducing carbon sources. **(A)** SDS-PAGE analysis and pNPC hydrolytic activities of the extracellular proteins from QM9414 (left panel) and P_*tcu1*_-*xyr1* (right panel) strains grown on 1% (wt/vol) glucose without exogenous addition of CuSO_4_. **(B)** The same as in **(A)**, but the strains were grown on 1% (wt/vol) glycerol. **(C)** Filter paper hydrolytic activity of the cellulases produced by P_*tcu1*_-*xyr1* on 1% (wt/vol) glycerol or those produced by QM9414 strain on 1% (wt/vol) Avicel or glycerol cultured with or without CuSO_4_. A significant difference (*P* < 0.05) was detected between the produced cellulases of P_*tcu1*_-*xyr1* on 1% (wt/vol) glycerol without CuSO_4_ and those of QM9414 on 1% (wt/vol) glycerol. **(D)** The extracellular pNPC hydrolytic activity of P_*tcu1*_-*xyr1* cultured on 1% (wt/vol) glycerol in the presence of the indicated concentrations of CuSO_4_. Equal amount of culture supernatant relative to biomass was measured for all the assays. Error bars are the SD from at least two biological replicates.

To further ask to what extent overexpression of *xyr1* alone would effect on launching the production of cellulases under non-inducing conditions, we then determined the synthesis of cellulases by P_*tcu1*_-*xyr1* on various other carbon sources. As shown in Figure 6A,B, the expression of *xyr1* in P_*tcu1*_-*xyr1* resulted in a constitutive expression of cellulases on either glucose or glycerol, whereas no cellulases could be detected for QM9414 under these conditions. Consistent with these results, significant extracellular pNPC and pNPG hydrolytic activities were detected for P_*tcu1*_-*xyr1* cultured without addition of copper, whereas hardly any such activities were observed when QM9414 was cultured under the same conditions (Figure 6A,B). Analysis of the hydrolytic activity toward filter paper revealed that significant activities were detected for cellulases produced by P_*tcu1*_-*xyr1* on glycerol over time, which was almost equivalent to cellulases produced by QM9414 induced with Avicel at 48 h (Figure 6C). Again, the cellulase production was inhibited with increasing amount of CuSO_4_ added to the culture, and BCS was able to partially recover the production after a complete inhibition was inflicted (Figure 6C,D). Altogether, the above results indicated that P_*tcu1*_-*xyr1*-mediated expression of *xyr1* is capable of achieving the full production of cellulases without induction.

**Figure 7:**
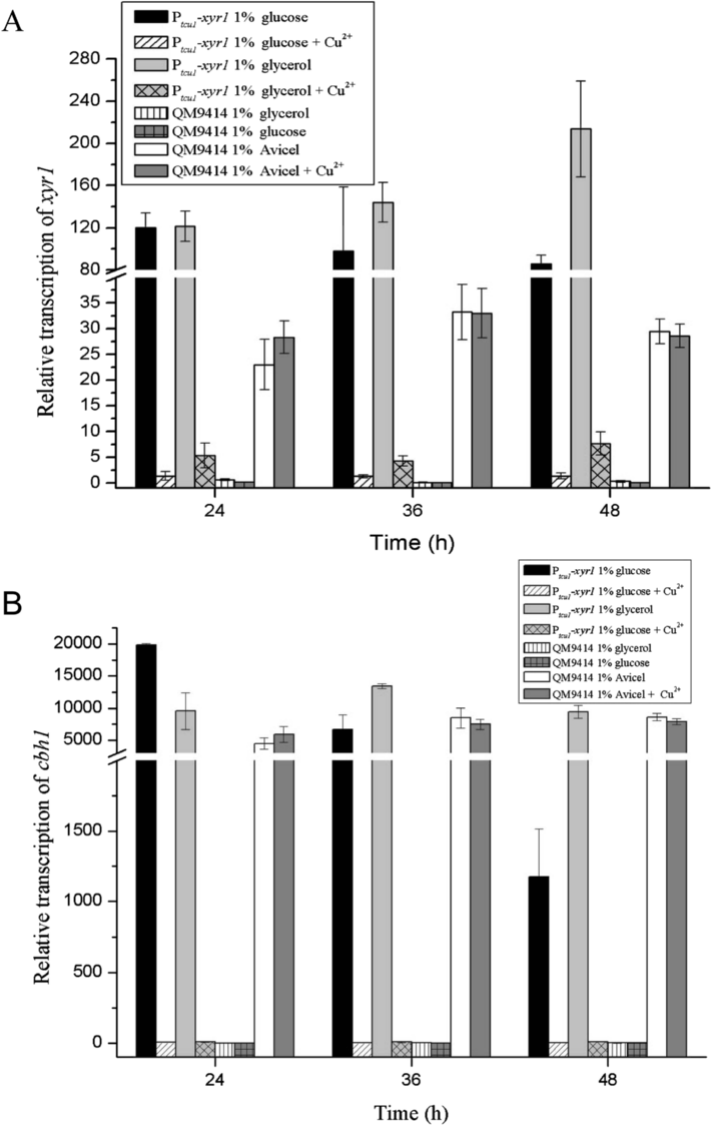
qRT-PCR analysis of the transcription of *xyr1* and *cel7a* in strains P_*tcu1*_-*xyr1* and QM9414. **(A)** Transcript abundance of *xyr1 of* P_*tcu1*_-*xyr1* on non-inducing carbon sources compared with those of QM9414 on Avicel in the presence or absence of 10 μM CuSO_4_. **(B)** Levels of *cel7a* mRNA in P_*tcu1*_-*xyr1* on non-inducing carbon sources compared with those in QM9414 on Avicel with or without addition of 10 μM CuSO_4_. Significant differences (*P* < 0.05) were detected for the transcription of *xyr1* between P_*tcu1*_ *-xyr1* on glucose or glycerol without CuSO_4_ and QM9414 on Avicel at all three indicated time points. For the transcription of *cel7a*, a significant difference (*P* < 0.001) was detected between P_*tcu1*_ *-xyr1* at 48 h on glucose and those in QM9414 on Avicel. Error bars are the SD from three biological replicates.

To test whether the deregulated expression of cellulases in strain P_*tcu1*_*-xyr1* was absolutely due to the overexpression of *xyr1*, the transcription of *xyr1* was analyzed on 1% glucose or glycerol. The results showed that, during cultivation on glucose or glycerol, the abundance of *xyr1* mRNA in P_*tcu1*_*-xyr1* remained constantly high after 24 h, which was three- to fivefold higher than that in QM9414 cultured on Avicel (Figure 7A). The relative transcript abundance of *cel7a* in strain P_*tcu1*_*-xyr1* was also found to display a high level that was largely comparable to that in QM9414 induced with Avicel though a slight reduction in the *cel7a* abundance was observed at 48 h of culture on glucose (Figure 7B).

## Discussion

*T. reesei* is well known for its high capacity to secrete large amounts of lignocellulosic enzymes. Whereas tremendous efforts have been made to continuously improve the performance of the native cellulase system of *T. reesei* either by optimizing existing enzymatic activities or increasing the production efficiency of cellulases, research has been also directed toward developing it into a heterologous host for large-scale protein production. Although several gene expression systems have been successfully used in *T. reesei* including those based on the inducible *cel7a* promoter as well as the constitutive promoters from glucolytic genes [8-10,18], alternatives to control gene expression by both activation and repression independent of the nutritional state of the culture are still desirable. In the present study, we developed a copper-controllable expression system based on the promoter of a putative copper transporter encoding gene. The system is highly responsive to environmental copper levels and can mediate the high-level expression of heterologous and homologous proteins. Importantly, the controllable expression does not seem to be influenced by the carbon sources used. Moreover, the growth and the cellulases induction are hardly affected no matter whether the exogenous addition of copper sulfate or not. Although we cannot exclude the possibility that the trace amount of copper in the medium even without exogenous addition still inflicts a repressive effect on P_*tcu1*_, our observation that the expression of *tcu1* was not significantly decreased by exogenous addition of 200 nM of copper but was almost abolished by concentrations above 500 nM copper sulfate, suggested that there might be a threshold of copper required to shut off the expression of *tcu1* probably through inactivating a homologous transcription activator CUF1p [19,20]. In contrast with a previous report [21], copper chelator BCS only partially relieved the repression of *tcu1* expression compared with that without any exogenous addition of copper. This discrepancy may lie in the differences either in the permeability of BCS in *T. reesei* and *N. crassa* or in the copper binding affinities of CUF1p. Regardless of this, the successful expression of the native cellulases by P_*tcu1*_ under non-inducing conditions demonstrates that the developed system provides a useful alternative to overexpress selected proteins including cellulases or their variants with relatively high purity and free of other cellulase contaminants. It should be noted though that CEL7A expressed under these conditions is almost inactive, suggesting that a proper folding into the active conformation may not be achieved. The precise mechanism warrants further study. As reported previously, the stringent control of gene expression as displayed by P_*tcu1*_ also provides a powerful tool to elucidate gene function in *T. reesei* [11].

Cellulase production in *T. reesei* is a highly coordinated process regulated by a suite of transcription factors with XYR1 being considered the main activator of cellulase/hemicellulase gene expression [4,5]. Regulation of *xyr1* expression has thus been proposed to have a significant impact on the production of various polysaccharide hydrolytic enzymes. Transcription of *xyr1* is kept at a very low level on D-glucose, whereas its expression is strongly upregulated under inducing conditions [7]. Consistent with the role of XYR1, it has been observed that the constitutive expression of *xyr1* results in a changed hydrolase expression profile and elevated extracellular hydrolytic enzyme activities [5,6,16]. The strongly elevated basal expression of *xyr1* has been also observed in a hyperproducer strain even before induction [7]. Moreover, it has reported that a single point mutation of XYR1, A824V, results in a highly elevated basal level of cellulase expression [22]. Further supporting the above assumption, we found that ectopic expression of *xyr1* driven by P_*tcu1*_ led to strongly increased production of cellulases on non-inducing carbon sources to a level comparable to that achieved under inducing conditions. Although the possibility cannot be excluded that other genetic traits may have been altered and contributed to the observed phenotype due to the random integrations of the *xyr1* cassette, the success to achieve the efficient cellulase production in the recombinant strain seems to rely on the overexpression of *xyr1* since the observed cellulase production is absolutely responsive to exogenous copper levels. Our results are also in accordance with a recent report that a strong increase in *xyr1* basal expression levels results in very high levels of CMCase activity during growth on glucose although no direct evidence regarding their relationship has been presented [23]. Whereas the exact mechanism of how the overexpressed XYR1 results in the deregulated production of cellulases warrants further study to provide a better understanding of cellulase formation, genetic engineering on the basis of the present strategy targeted other transcription factors holds great potential of further improving cellulase production in a more straightforward way in *T. reesei*.

## Conclusion

We have developed a protein expression system based on the promoter of a copper transporter (P_*tcu1*_) in *T. reesei*, the usefulness of which was illustrated by the high-level expression of specific cellulases and GFP when cultivated on D-glucose or glycerol as the sole carbon source. Meanwhile, full production of cellulases was achieved by P_*tcu1*_-mediated expression of XYR1 even under the non-inducing conditions, which allowed us to better understand the mechanism of cellulase production and further improve the production of cellulases by *T. reesei*.

## Methods

### Strains, medium, and culture conditions

*T. reesei* QM9414 (ATCC 26921), a cellulase-enhanced mutant derived from the original strain QM6a, was used as a control strain for analysis of cellulase production. Strain Δ*xyr1* derived from QM9414 was kindly provided by Dr. Robert L. Mach [24]. The Δ*cel7a* strain was constructed as previously described [25]. Strains were grown in 1-l Erlenmeyer flasks on a rotary shaker (250 rpm) at 30°C in the medium described by Mandels and Andreotti with respective carbon sources added at a final concentration of 10 g/l [26]. *Escherichia coli* DH5α was used for routine plasmid construction and amplification.

For transcription and cellulase production analysis, *T. reesei* was precultured on glycerol for 48 h and grown for another 12 h in the same fresh medium. Mycelia were harvested by filtration and washed twice with medium with no carbon source. Equal amounts of mycelia were transferred to a fresh medium containing 10 g/l of corresponding carbon sources without peptone, and the incubation was continued for the indicated time periods.

### Plasmid construction

The 1715-bp fragment comprising the promoter of the *tcu1* gene (jgi:Trire2:52315) was amplified from the genomic DNA of strain QM9414 and subsequently ligated into pMD19-T to generate pMDP*tcu1*. After digestion with *Not*I and *Hin*dIII, the *trpC* terminator was inserted into pMDP_*tcu1*_ to obtain pMDP_*tcu1*_*-T*_*trpC*_*.* The 2.7-kb *pyr4* gene fragment was amplified from pFG1 [27] and digested with *Hin*dIII and *Sal*I, and was incorporated into pMDP_*tcu1*_*-T*_*trpC*_ to obtain pMDP_*tcu1*_*-T*_*trpC*_*-pyr4*.

To construct the plasmids for respective expression of *cel7a* and *cel7b* driven by the promoter of *tcu1*, the coding sequences of *cel7a* and *cel7b* were amplified from the genomic DNA of QM9414, respectively, digested with *Asc*I and *Spe*I, and then ligated into pMDP_*tcu1*_*-T*_*trpC*_*-pyr4* to obtain pMDP_*tcu1*_*-cel7a-T*_*trpC*_*-pyr4* and pMDP_*tcu1*_*-cel7b-T*_*trpC*_*-pyr4*. To construct the expression plasmid for *gfp*, the coding sequence of *gfp* was amplified from pIG1783 [28], digested with *Pme*1 and *Spe*I, and subsequently ligated into pMDP_*tcu1*_*-T*_*trpC*_ to obtain pMDP_*tcu1*_*-gfp-T*_*trpC*_*.* Similarly, the coding sequence of *xyr1* was amplified with cDNA of QM9414 as the template, digested with *Asc*1 and *Spe*I, and then incorporated into pMDP_*tcu1*_*-T*_*trpC*_ to generate the expression plasmid for *xyr1*, pMDP_*tcu1*_*-xyr1-T*_*trpC*_.

### Construction of recombinant *T. reesei* strains

Fungal transformation was performed as described by Penttila *et al*. [29]. Constructed plasmids of pMDP_*tcu1*_*-cel7a-T*_*trpC*_*-pyr4* and pMDP_*tcu1*_*-cel7b-T*_*trpC*_*-pyr4* were transformed into Δ*cel7a* strain to obtain recombinant strains of P_*tcu1*_-*cel7a* and P_*tcu1*_-*cel7b*, respectively. The plasmid of pMDP_*tcu1*_*-gfp-TtrpC* was co-transformed into *T. reesei* QM9414 with pRLMex-30 to obtain the recombinant strain of P_*tcu1*_-*gfp*. Similarly, pMDP_*tcu1*_*-xyr1-T*_*trpC*_ was co-transformed with pRLMex-30 into strain Δ*xyr1* to obtain the recombinant strain of P_*tcu1*_-*xyr1*. Transformants of P_*tcu1*_-*cel7a* and P_*tcu1*_-*cel7b* were selected on minimal medium for uridine prototroph, and transformants of P_*tcu1*_-*gfp* and P_*tcu1*_-*xyr1* were selected for resistance to 100 μg/ml of hygromycin. All the recombinant strains were verified for the presence of the entire expression cassette by PCR.

### Fluorometric analysis

The fluorescence of the mycelium of P_*tcu1*_*-gfp* was detected with a Nikon Eclipse 80i fluorescence microscope (Nikon, Melville, NY, USA), and images were captured and processed with the NIS-ELEMENTS AR software program. For fluorescence quantification of GFP expression driven by the promoter of *tcu1* in the presence of various concentrations of copper in liquid culture, strain P_*tcu1*_*-gfp* was precultured on Mandels-Andreotti medium with glycerol, transferred into Mandels-Andreotti medium with 1% glucose plus 0 to 10 μM CuSO_4_, and harvested for 60 h. After being washed with 0.9% NaCl, the mycelia were suspended 50 mM HEPES buffer at pH 7.4 and grinded with micro glass beads by Precellys homogenizers (Bertin Technologies, Montigny-le-Bretonneux, France). Cell debris was removed by centrifugation at 14,000 × *g* for 10 min, and 200 μl of the supernatant was used to determine the GFP fluorescent intensity with a 96-well spectrofluorometer at an excitation wavelength of 485 nm and an emission wavelength of 535 nm. The protein concentration in the supernatant was determined using the method of the Bradford protein assay with bovine serum albumin (BSA) as the standard, and the relative fluorescent intensity was calculated according to an equation as follows:

The relative fluorescent intensity = [measured fluorescent intensity/protein concentration]/[background fluorescent intensity of QM9414/background protein concentration of QM9414].

### Quantitative RT-PCR

Total RNAs were extracted using TRIzol reagent (Invitrogen, Grand Island, NY, USA) and purified using the TURBO DNA-free kit (Ambion, Austin, TX, USA) to remove DNA according to the manufacturer’s instructions. Reverse transcription was carried out using the PrimeScript RT reagent Kit (Takara, Tokyo, Japan) according to the instructions. Quantitative PCR was performed on a Bio-Rad myIQ2 thermocycler (Bio-Rad, Richmond, CA, USA). Amplification reactions were performed using the SYBR Green Supermix (Takara, Tokyo, Japan) according to the instructions. Data analysis was performed using the relative quantitation/comparative CT (△△CT) [30]. The endogenous *actin* gene was used as a normalized control gene. For analysis of *tcu1* transcription, *tcu1* mRNA level under the most repressed conditions was set to 1 and other points showed the fold of difference relative to these most repressed conditions. And for cellulase gene expression, mRNA level in uninduced conditions (glucose or glycerol) was set to 1 and other time points showed the fold of difference relative to this uninduced sample. At least three biological replicates were carried out for each experiment.

### Enzyme activity measurements

Cellobiohydrolase and β-glucosidase activities were determined with 2 mM *p*-nitrophenol-D-cellbioside (Sigma-Aldrich, St. Louis, MO, USA) plus 0.1% δ-gluconolactone and 5 mM *p*-nitrophenyl-β-D-glucopyranoside (Sigma-Aldrich, St. Louis, MO, USA) as substrates at 45°C, respectively. The assays were carried out in 200 μl of reaction mixture containing 50 μl of culture supernatant and 50 μl of the respective substrate plus 100 μl of 50 mM sodium acetate buffer (pH 4.8) with incubation at 45°C for 30 min. The assay for endoglucanase activity measurement was carried out in 100 μl of reaction mixture containing 50 μl of culture supernatant and 50 μl of 0.5% CMC in 50 mM sodium acetate buffer (pH 4.8) and incubated at 50°C for 30 min. The total cellulase activity assay was carried out in 200 μl of reaction mixture including 50 μl of culture supernatant and 150 μl 50 mM sodium acetate buffer (pH 4.8) with a Whatman No. 1 filter paper strip as the substrate and incubated at 50°C for 1 h. The reducing sugars were determined spectrophotometrically by using 3,5-dinitrosalicylic (DNS) acid method with glucose as standards. At least two biological replicates were carried out for each experiment.

### Protein analysis

SDS-PAGE and Western blotting were performed according to standard protocols [31]. The cellobiohydrolase CEL7A was detected by immunoblotting using a polyclonal antibody raised against amino acids (426 to 446) of CEL7A as previously described [32].

## Abbreviations

BCS: bathocuproinedisulfonic acid
BSA: bovine serum albumin
CCR: carbon catabolite repression
CMC: carboxymethylcellulose
FPA: filter paper activity
GFP: green fluorescent protein
pNPC: *p*-nitrophenol-D-cellbioside
pNPG: *p*-nitrophenyl-β-D-glucopyranoside
P_*tcu1*_: promoter of a copper transporter encoding gene *tcu1* in *T. reesei*
XYR1: xylanase regulator 1
